# Encyclopedia of bacterial gene circuits whose presence or absence correlate with pathogenicity – a large-scale system analysis of decoded bacterial genomes

**DOI:** 10.1186/s12864-015-1957-7

**Published:** 2015-10-13

**Authors:** Maksim Shestov, Santiago Ontañón, Aydin Tozeren

**Affiliations:** School of Biomedical Engineering, Science and Health Systems, Drexel University, Philadelphia, PA USA; College of Computing and Informatics, Drexel University, Philadelphia, PA USA

**Keywords:** Bacteria, Pathogen, Ortholog, Gene circuits, Virulence factors

## Abstract

**Background:**

Bacterial infections comprise a global health challenge as the incidences of antibiotic resistance increase. Pathogenic potential of bacteria has been shown to be context dependent, varying in response to environment and even within the strains of the same genus.

**Results:**

We used the KEGG repository and extensive literature searches to identify among the 2527 bacterial genomes in the literature those implicated as pathogenic to the host, including those which show pathogenicity in a context dependent manner. Using data on the gene contents of these genomes, we identified sets of genes highly abundant in pathogenic but relatively absent in commensal strains and vice versa. In addition, we carried out genome comparison within a genus for the seventeen largest genera in our genome collection. We projected the resultant lists of ortholog genes onto KEGG bacterial pathways to identify clusters and circuits, which can be linked to either pathogenicity or synergy. Gene circuits relatively abundant in nonpathogenic bacteria often mediated biosynthesis of antibiotics. Other synergy-linked circuits reduced drug-induced toxicity. Pathogen-abundant gene circuits included modules in *one-carbon folate*, *two-component system*, *type-3 secretion system*, and *peptidoglycan* biosynthesis. Antibiotics-resistant bacterial strains possessed genes modulating phagocytosis, vesicle trafficking, cytoskeletal reorganization, and regulation of the inflammatory response. Our study also identified bacterial genera containing a circuit, elements of which were previously linked to Alzheimers disease.

**Conclusions:**

Present study produces for the first time, a signature, in the form of a robust list of gene circuitry whose presence or absence could potentially define the pathogenicity of a microbiome. Extensive literature search substantiated a bulk majority of the commensal and pathogenic circuitry in our predicted list. Scanning microbiome libraries for these circuitry motifs will provide further insights into the complex and context dependent pathogenicity of bacteria.

**Electronic supplementary material:**

The online version of this article (doi:10.1186/s12864-015-1957-7) contains supplementary material, which is available to authorized users.

## Background

Microbiology experiments identified a large number of bacterial virulence mechanisms conserved through evolution [1]. Pathogenic bacterial strains could be defined as those with capacity to harm the host and cause disease [2]. A bacterial strain may appear as asymptotic or pathogenic depending on the state of the immune system of the host [3], composition of the microbiome [4], presence and absence of elicitors [5], and other environmental factors [6]. Synthesis of secreted virulence factors are transcriptionally regulated by environmental stimuli [7]. Production of enzymes that degrade host cytoskeleton and cause damage is linked to bacterial strain density and quorum sensing [8]. It is clear that pathogenicity is context dependent.

The large research literature on bacterial pathogenicity was recently curated into an open access web platform, the Virulence Factor Database or VFDB [2, 6, 9]. The database presents genes and gene groups (virulence factors) associated with infectious disease. Since the metagenome of a human microbiome contains over three million genes, the extent of our knowledge on virulence genes may not be close to saturation. Nevertheless, not only the VFDB database is valuable for identifying genes linked to pathogenicity in specified bacteria (even strains), but it also serves as a benchmark for studies predicting pathogenicity linked gene clusters.

Virulence factors may cause disease in multiple hosts [1]. Yet, specificity of virulence factors to certain bacterial and strains is poorly understood. A recent study involving 50 bacterial genomes found some virulence factors to be exclusive to pathogenic bacterial strains in this small sample [10]. Virulence genes abundant in pathogenic as well as nonpathogenic strains included those facilitating coding of translocation proteins, apparatus proteins, and chaperons. The findings of the study suggest the presence of pathogenic gene circuits in which some genes belong exclusively to pathogenic strains whereas others can also be abundant in bacterial strains synergistic or commensal to the host.

The drivers of pathogenicity may not only be virulence factors but also the absence of antivirulence factors in bacterial genomes [11–13]. Some bacterial species contain strains known as commensal to a host and other strains that exhibit pathogenicity. Transformation to pathogenic state maybe due to exchange of DNA fragments between bacteria [14]. Literature points out to the acquisitions of gene clusters and pathogenicity islands via horizontal gene transfer [15–17]. Deletion of genes or loss of gene function through mutation appears to be part of the adaptation to newly acquired pathogenesis [18].

Recent identification of the elicitors for activating antibiotics-synthesizing bacterial gene circuits [5] provides a new dimension in our understanding of the environmental forces affecting pathogenicity. Although, the research literature contains many examples of bacterial genes linked to synthesis of antibiotics [19–21], a large-scale study exposing diversity of such genes and gene circuits is yet to be carried out. The same is true for pathogenic gene circuits. That is the reason we set out in this study a systematic approach to identify and annotate prokaryotic ortholog gene circuits whose presence or absence are linked to phenotypes of antagonism to the host. Comparative genomics has been utilized before in the identification of drug and vaccine targets in Staphylococcus aureus [22] and mycobacterial peptidoglycan remodeling enzymes linked to pathogenicity [23]. Ours, however, takes it one step further, employing comparisons among 2527 distinct genomes. Restriction of genome comparison to ortholog groupings reduced dimension of this meta-scale analysis. It also expanded the reach of findings within the context of evolution. Aspects of pathogenicity, which are universal across eukaryotic hosts, will likely emerge in this approach [24].

Our methodology has multiple steps. First, we annotate existing genome sequences of bacterial strains as pathogenic or otherwise, based on literature curation. Next, we identify the presence and absence of orthologs in the genomes of these bacterial strains. Thirdly, we determine the relative abundance or absence of these orthologs in the pathogenic and non pathogenic strains within and across genera. Projection onto cellular pathways result in annotation of gene circuits linked to pathogenicity. Extensive comparison with experimental literature provides biological context to our findings. Our study in effect creates an encyclopedia for pathogenicity, built on big data on genome sequences and literature on phenotypes of bacterial strains.

## Results

### A. Orthologs linked either to pathogenicity or synergy with the host

#### Annotation of pathogenic bacterial strains

Our literature search detailed in the Methods section identified 949 bacterial strains as pathogenic, meaning they had been reported in the literature as pathogenic to an animal host at least once. The label pathogenic, in the way we use it, doesn’t mean that the bacterial strain will cause disease to an animal host under all circumstances.

Additional file 1 presents the list of bacterial strains deemed as pathogenic, with evidence provided in the file in the form of references or database citations. This supplemental file also contains labeling of pathogenic strains found as antibiotic resistant in the Antibiotic Resistance Genes Database (*ARDB)* [25]. Table 1 shows a sample of the bacterial genera possessing both pathogenic and nonpathogenic strains. Strains of the same bacterial genus often separated into pathogenic and nonpathogenic clusters.

**Table 1:** List of bacterial genera highlighting the number of strains that have been associated with the pathogenic state, as well as the strains associated with the commensal state in the present study

Total bacterial strain genomes					2527
Total pathogenic bacterial strains					949
KEGG based pathogenic strains					767
Additional literature based pathogenic strains					182
Genus	Total	Pathogenic	Genus	Total	Pathogenic
Streptococcus^a^	120	77	Rickettsia^a^	41	19
Chlamydia^a^	98	81	Burkholderia^a^	39	17
Bacillus^a^	81	22	Listeria^a^	34	25
Candidatus^a^	71	4	Campylobacter^a^	26	13
Mycobacterium^a^	65	34	Vibrio	25	20
Mycoplasma^a^	65	54	Brucella	20	18
Escherichia^a^	64	39	Acinetobacter	19	12
Helicobacter^a^	63	47	Francisella	19	13
Pseudomonas^a^	53	12	Yersinia	19	17
Staphylococcus^a^	51	42	Borrelia	18	14
Corynebacterium^a^	50	34	Neisseria	18	18
Clostridium^a^	49	22	Treponema	17	13
Salmonella^a^	45	36	Haemophilus	16	13

The “^a^” identifies those genera for which we have also conducted within genus comparisons

There was no KEGG- or literature-recorded evidence of pathogenicity for the remaining 1578 decoded bacterial strain genomes in the KEGG database. Thus, they were deemed for this study as nonpathogenic. Additional file 2 presents the list of the 1578 bacterial strains deemed nonpathogenic in our study.

#### Orthology contents of pathogenic and nonpathogenic strains

Next, we created two sets of matrices for ortholog genes, one set for the genomes of pathogenic and the other for nonpathogenic strains. The columns of each matrix identified the bacterial strain whereas rows identified whether an ortholog was present (1) or absent (0) in that strain. It turned out that 7194 different orthologs were present in at least one of the genomes of the 2527 bacterial strains under study. These large matrices are used for the abundance computations presented in this study and hence included as Additional file 3.

**Fig. 1:**
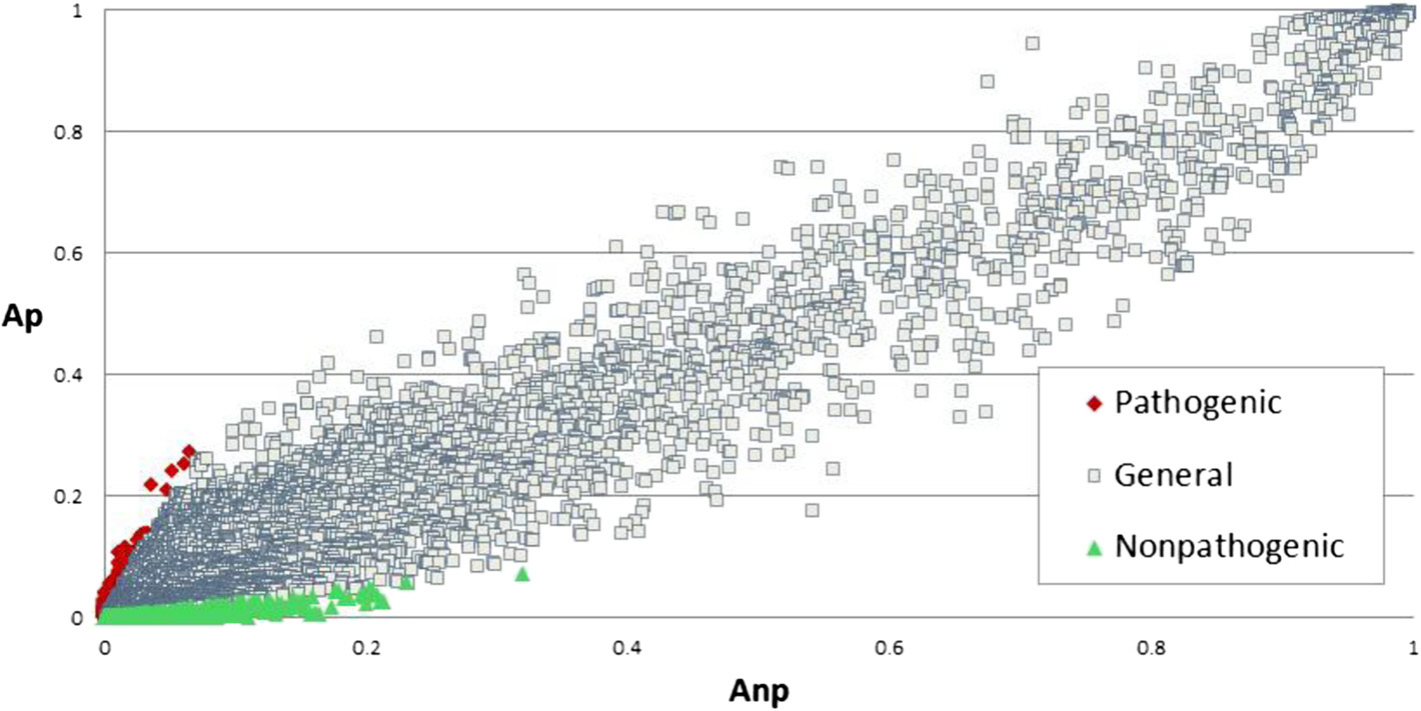
Scatter diagram of relative abundance of 7194 orthologs found in 2527 decoded bacterial genomes. The horizontal axis represents the percentage of nonpathogenic strains presenting the ortholog (*Anp*) whereas the vertical axis represents the corresponding percentage in pathogenic strain subpopulation (*Ap*). The pathogen-abundant (*PA* > 4) and nonpathogen-abundant orthologs (*PA* < 0.25) were marked in red and green, respectively. Note that *PA* = *Ap/Anp*

For a given ortholog, fractions of pathogenic and nonpathogenic bacterial strains presenting the ortholog in their genomes are represented by symbols *Ap* and *Anp*, respectively. The scatter diagram shown in Fig. 1 presents the *Ap* and *Anp* values for the 7194 orthologs present in bacterial strains. It appears that most of the orthologs have comparable presence in all bacterial strains whereas a small portion (green and red dots for pathogenic and nonpathogenic strains, respectively) is biased towards one of the two phenotypes.

**Fig. 2:**
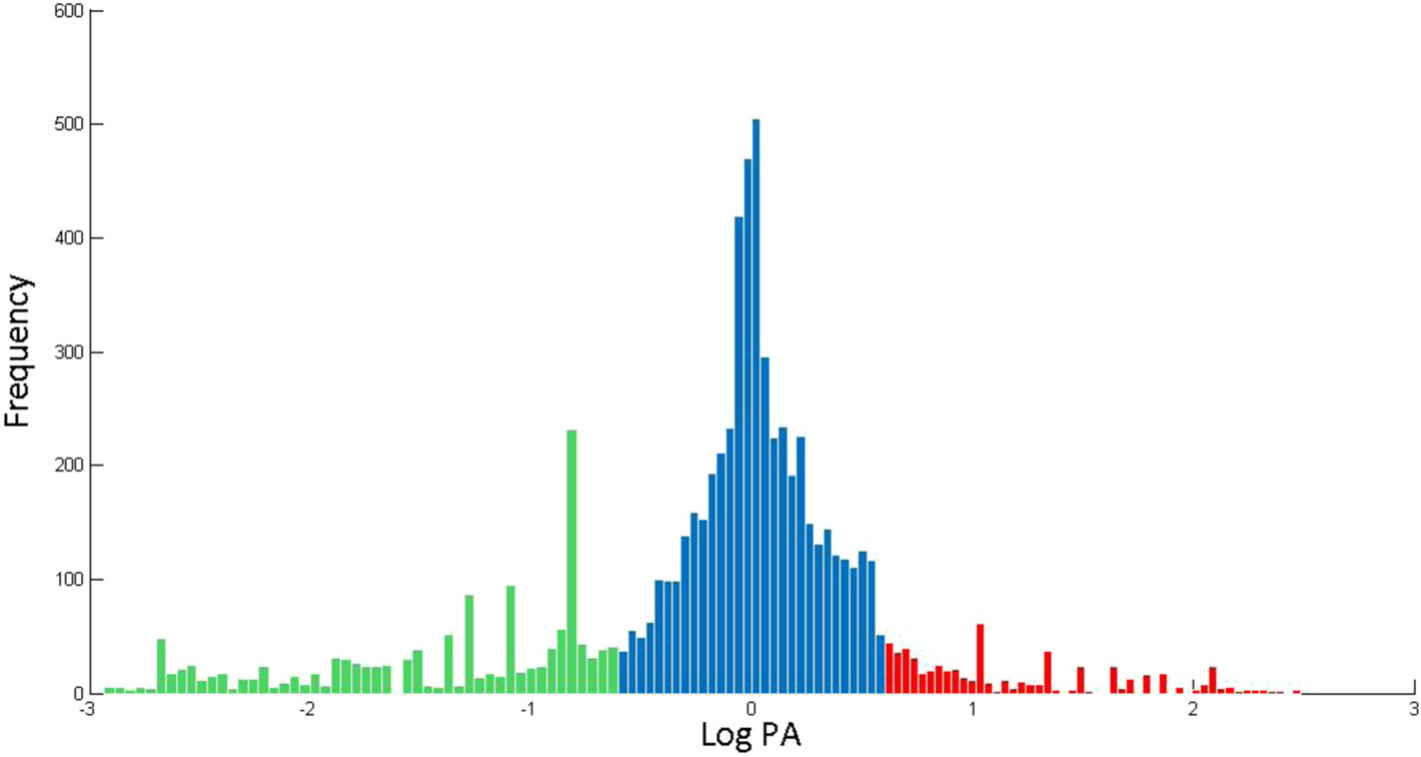
Histogram showing the frequency of occurrence of orthologs with respect to the pathogen abundance score (*PA*). The two edges of the histogram (*PA* > 4, *PA* < 0.25) are marked in red and green, respectively

The histogram shown in Fig. 2 is another view of the data presented in the scatter diagram in Fig. 1. Here, we plotted the frequency of occurrence against the pathogen abundance score *log PA* for all the orthologs under consideration. The parameter *PA* = *Ap /* (*Anp* + 0.0001) is a measure of relative abundance of the ortholog in pathogenic strains. In cases where *Anp* equaled zero, the equation still enables division due to the presence of 0.0001 in the denominator. The two tail ends of the distribution indicate those orthologs abundant in pathogenic but rarely found in nonpathogenic (red) and vice versa (green). The cutoff values we used (*PA > 4,* and *PA < ¼*), although somehow arbitrary, were placed at the inner edges of the tails of the histograms.

**Fig. 3:**
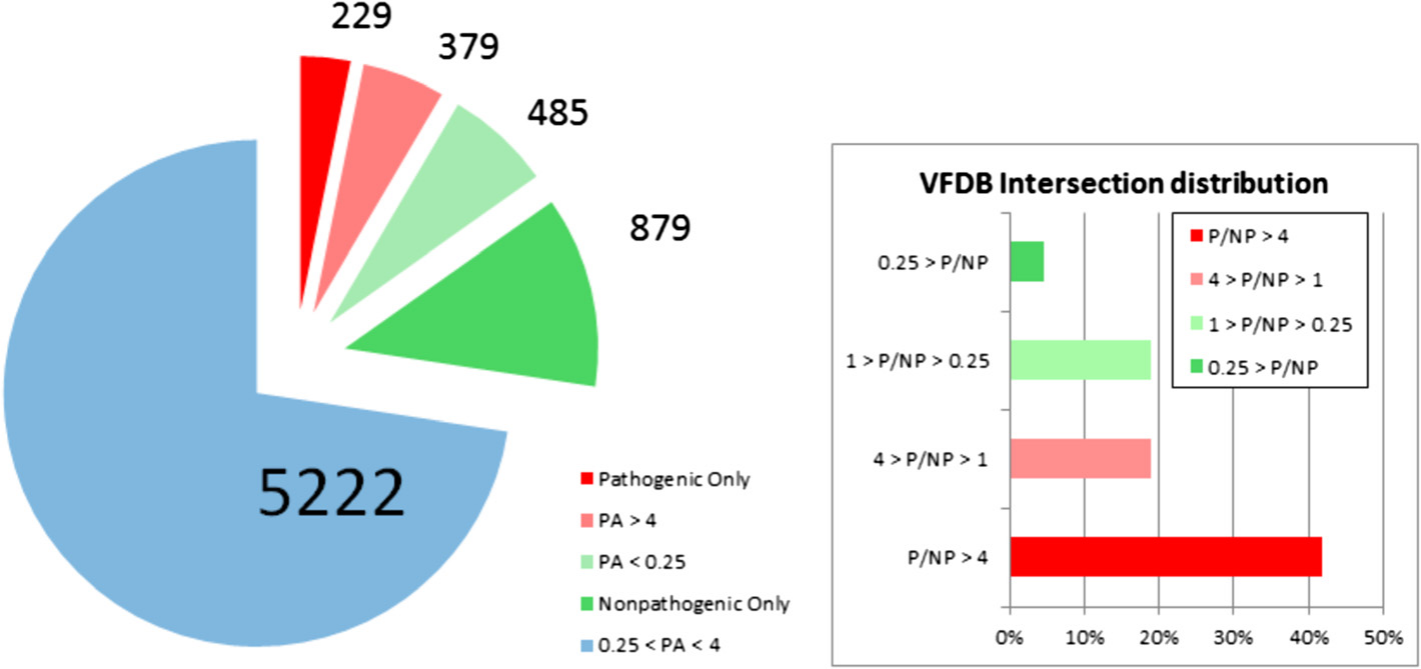
Pie charts indicating the distribution of orthologs of the present study with respect to ortholog abundance (3A) and the virulence factors presented by the VFDB web platform (3B) as a function of the pathogen abundance score *PA*

Shown in Fig. 3a are the overall characteristics of the PA distribution among the orthologs. In brief, there were 229 pathogenic only and an additional 379 orthologs for which *PA > 4,* representing about 8 percent of the orthologs found in our bacterial strain library*.* Taken together, we deemed this group as pathogen abundant or pathogen-linked. Total number of genes in the 608 pathogen-abundant orthologs was 18,982, indicating their presence in a diverse set of bacterial species. Pathogen exclusive orthologs comprised only 1,518 of this set of genes, suggesting most genes previously linked to pathogenicity is not exclusive to disease-causing bacterial strains.

The number of orthologs exclusive to nonpathogenic strains was much larger at 879, and an additional 485 had *PA < ¼*. The rest, a total of 5222 orthologs, were commonly present among pathogenic and nonpathogenic strains. It is expected that these numbers will change as the number of available bacterial genomes in the literature increase from thousands to tens of thousands.

Next, we identified those orthologs in our list, a total of 1308, which were also present in the Virulence Factor Database, *VFDB,* by matching either the gene names or gene descriptions. As indicated in Fig. 3b, VFDB orthologs are significantly biased towards pathogen abundant orthologs. Additional file 4 lists the pathogen-abundant and non-pathogen-abundant orthologs in accordance with the *PA* ranking, along with VFDB labeling if present in that database. Overall, our study indicates the absence of one-to-one match between known virulence factors and pathogen-abundant orthologs.

#### Orthologs enriched in pathogenic, antibiotic resistant, and nonpathogenic bacterial strains

**Fig. 4:**
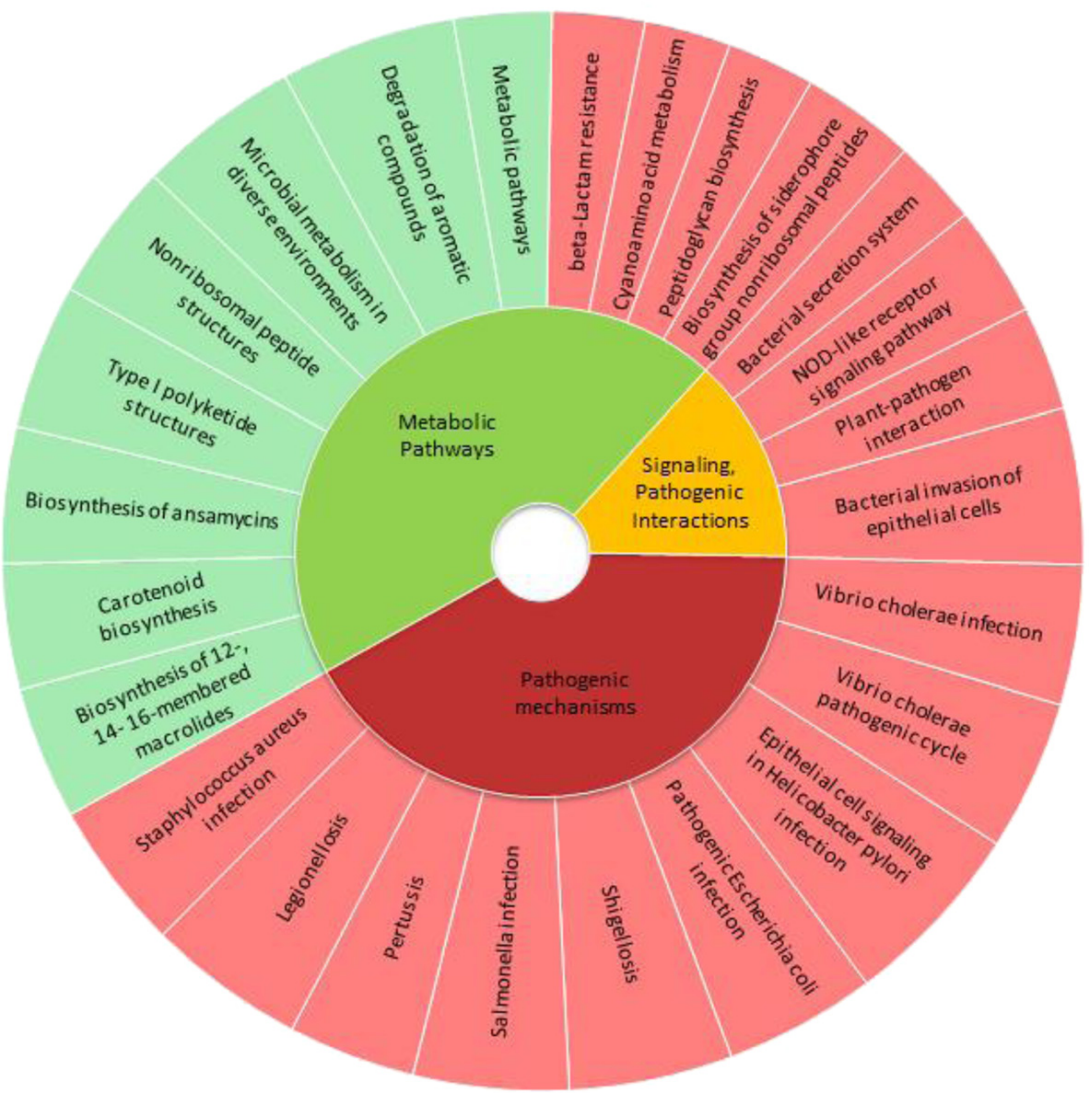
Pie chart for statistical enrichment of KEGG pathways by pathogen-abundant (*PA* > 4, red) and nonpathogen-abundant (*PA* < 0.25, blue) orthologs, respectively

Statistical enrichment of KEGG pathways was conducted based on the hypergeometric test for ortholog sets *PA > 4,* and *PA < ¼*, respectively. Results are shown in Fig. 4. Orthologs abundant in pathogenic strains crowd pathogen-linked cellular pathways: *Staphyloccus aureus*, *Leigonellosis*, *Pertussis*, *Salmonella*, *Shigellosis*, and *Escherichia coli* infections, as well as epithelial signaling in *H. pylori* infection. Pathogen abundant orthologs are also found in pathways involving bacterial secretion systems, Nod like receptor signaling, bacterial invasion of epithelial cells, and plant-pathogen interactions.

Orthologs found exclusively in nonpathogenic strains occupy nodes in metabolic pathways (Fig. 4). These pathways include biosynthesis of *peptidoglycans*, *microlides*, *carotenoids*, a*nsamycins*, and *nonribiosomal* peptides. The GO cell compartment investigations not shown in the figure indicate that pathogen abundant orthologs are enriched in crosstalk positions of contact with the host whereas nonpathogen exclusive orthologs code proteins involving in events in the cell interior.

**Fig. 5:**
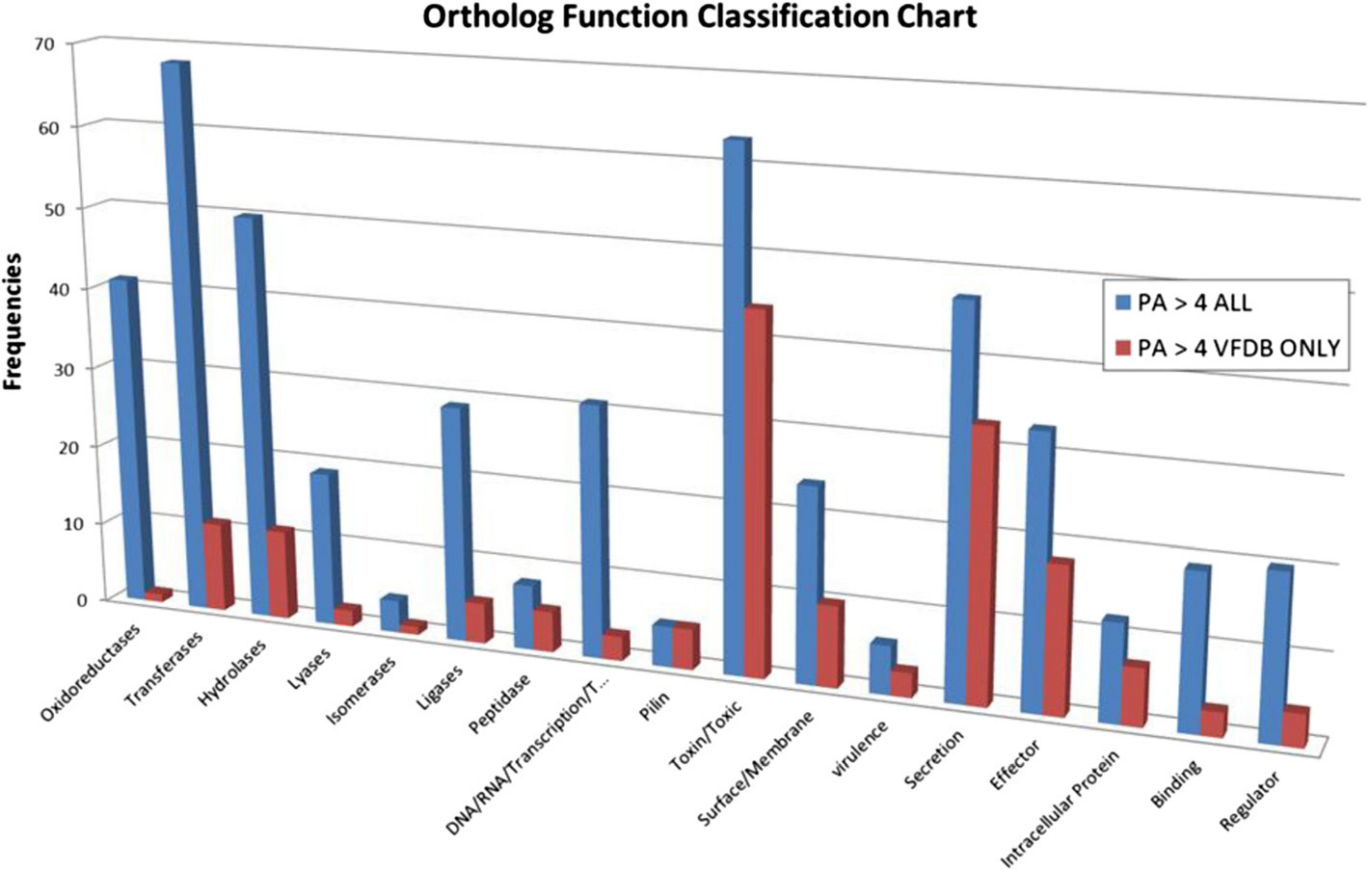
Gene ontology molecular function distributions in pathogen-abundant orthologs (*PA > 4*, blue) and in *VFDB* (red), respectively

Next, we looked at molecular function enrichments of pathogenic associated orthologs and compared the results with corresponding enrichments obtained using the VFDB database. Figure 5 shows that both our annotation and VFDB contain roughly equal amounts of orthologs in secretion, toxins, peptidase, and *pilin* categories. However, pathogen abundant list of the present study has significantly more abundance in enzyme categories such as *oxidoreductases*, *transferases*, *hydrolases*, *lyases*, and l*igases*. Some of the orthologs in our list are also enriched in regulatory function, particularly involving transcription and translation.

Pathway enrichment was also conducted within the population of pathogenic strains for the subset identified as antibiotic resistant using *ARDB* Database [25]. The hypergeometric test revealed the pathways shown in Table 2 as particularly enriched in antibiotic resistant strains. These included sphingolipid metabolism, producing bioactive metabolites that regulate cell function [26], PI3K-Akt signaling pathway, an intracellular pathway important in apoptosis [27], and Aminoacyl-tRNA biosynthesis [28]. Some of the modules in the enriched pathways also appear in eukaryotic processes for drug resistance against chemotherapy. One must caution, however, that the results could potentially change with the updating of ARDB, even if the p values in these enrichments are vanishingly small.

**Table 2:** KEGG reference pathways, which are statistically enriched by orthologs abundant in antibiotics-resistant bacteria. Hypergeometric test assumes as background the set of pathogen-linked orthologs.

Pathway	p value via hypergeometric test
D-Arginine and D-ornithine metabolism	0.00572
Amino sugar and nucleotide sugar metabolism	0.0273
Peptidoglycan biosynthesis	0.00135
Sphingolipid metabolism	0.00524
Aminoacyl-tRNA biosynthesis	0.00076
Phosphatidylinositol signaling system	0.04909
PI3K-Akt signaling pathway	0.00384
Amoebiasis	0.03099

### B. Gene circuits linked either to pathogenicity or synergy

This section presents results on genetic circuits statistically enriched in pathogenic and nonpathogenic bacteria. We used two different types of comparison to achieve our results: a) analyzing the entire set of genomes partitioned into pathogenic and nonpathogenic phenotypes; and b) conducting the same operation within genera for the 17 genera identified in Table 1 with a star. In the first approach, we mapped the list of ortholog genes linked to pathogenicity (PA > 4) and non-pathogenicity onto KEGG reference pathways and identified, based on KEGG repository, those multiply connected clusters of genes (gene circuits) containing at least three pathogen-linked or nonpathogen-linked orthologs. Results are shown in Tables 3 and 4, respectively. Additional file 5 presents corresponding results for within-genera comparisons, both for pathogen- and nonpathogen-linked circuits along with the genera containing such circuits.

**Table 3:** Gene circuits linked to pathogenic phenotype. Gene symbols in the table indicate orthologs in the circuit, which are abundant in pathogenic but rarely found in nonpathogenic strains

	Pathway	Circuit	Orthologs	Genera
1	Two Component system	Mg2+ starvation, antimicrobial peptide	pagC, pagO, pagD, pagK, pgtE	S. enterica*, B. aphidicola, E. coli*, P. ananatis, Y. pseudotuberculosis
2	Two Component system	Hexose phosphate uptake	uhpC, uhpA, uhpT	C. trachomatis, E. coli*, S. enterica*, S. aureus, L. monocytogenes
3	Two Component system	Competence Stimulating Peptide	ABCC-BAC.COMA, comA, comB, comC, comD, comE	S. pneumoniae, L. casei, L. rhamnosus, S. equi, L. plantarum
4	Two Component system	Hypoxia,Oxygen, Nitrogen asssimilation	devS, nreA, nreB, narT	S. aureus*, M. tuberculosis, M. bovis, M. canettii, P. polymyxa
5	Two Component system	Phosphpglycerate transport	pgtC, pgtB, pgtA, pgtP	S. enterica*, C. jejuni, V. cholerae, K. pneumoniae, E. coli
6	Biosynthesis of siderophore group nonribosomal peptides	Yersiniabactin	irp1, HMWP1, irp5, ybtE, irp3, ybtU	E. coli* , Y. pestis, R. solanacearum, C. diphtheriae, K. pneumoniae
7	Biosynthesis of siderophore group nonribosomal peptides	Pyochelin	pchF, pchG, pchD, pchE	P. aeruginosa, B. pseudomallei, B. cenocepacia, A. dieselolei, A. lipoferum
8	Bacterial secretion system	Type III	yscF, yscO, yscP, yscX, yscC, yscW	C. trachomatis*, S. enterica, E. coli*, C. psittaci, P. aeruginosa
9	Bacterial secretion system	Type V	vacA, yadA, yadB_C	H. pylori*, Y. pestis, Y. pseudotuberculosis, Y. enterocolitica, H. cetorum
10	Peptidoglycan biosynthesis	Peptidoglycan branch	sgtA, sgtB, femA, pbpA, femB,	S. aureus, C. Arthromitus, E. sp., S.
11	Carotenoid biosynthesis	Terpenpid backbone to Staphyloxanthin	crtM, crtP, crtQ, K10212, crtO	S. aureus*, B. megaterium, C. sp., S. lugdunensis, B. infantis
12	Salmonella infection	Translocon	sipB, ipaB, bipB, sipC, ipaC, bipC, sipD, ipaD, bipD, sseB, sseC, sseD	S. enterica*, B. pseudomallei, E. coli*, S. baltica, B. mallei
13	Salmonella infection	Type III secretion system/SPI-2 encoded	sseJ, sifA, sseF, sseG, pipB2, sspH2, sseI, srfH, spvB	S. enterica*, S. baltica, E. tarda, Y. enterocolitica, C. violaceum
14	Pentose phosphate pathway	D-Glucosaminate metabolism	dgaA-dgaE, PTS-Dga-EIIA, PTS-Dga-EIID, PTS-Dga-EIIB, PTS-Dga-EIIC	S. enterica*, E. coli*, E. faecalis, C. difficile, L. casei
15	Bacterial invasion of epithelial cells	ECM-receptor interaction	pfbA, sfb1, fnbA, fnbB	S. aureus, S. pyogenes, S. pneumoniae, S. dysgalactiae, S. equi
16	Phosphotransferase system	Sorbose to Sorbose 1-phosphate	PTS-Sor-EIIC, sorA, PTS-Sor-EIID, sorM, PTS-Sor-EIIA, sorF, PTS-Sor-EIIB, sorB	E. coli*, K. pneumoniae, L. casei, L. rhamnosus, S. flexneri
17	Phosphotransferase system	D-Glucosaminate to D-Glucosaminate 6-P	PTS-Dga-EIIC, dgaA-dga-D, PTS-Dga-EIIA, PTS-Dga-EIID,, PTS-Dga-EIIB	S. enterica*, E. coli*, E. faecalis, C. difficile, L. casei
18	Amino nucleotide sugar metabolism	CMP-Pse metabolism	pseC, pseH, pseF, UAP1	C. trachomatis, H. pylori*, C. jejuni, C. psittaci, P. acnes
19	Plant-pathogen interaction	Bacterial secretion system	hopAB, avrPtoB, avrPto1, avrRpm1, avrXccC, avrB	P. syringae, _., X. campestris, _.
20	Bacterial invasion of epithelial cells	Type III - Salmonella	sipA-sipD, ipaA-ipaD, bipD, bipC, bipB, sopD, sptP, sopE, ipgD, sopB	S. enterica, B. pseudomallei, E. coli, B. mallei, B. thailandensis
21	Bacterial invasion of epithelial cells	Type III - Shigella	sipC, ipaA-ipaC, bipC, sipB, bipB, ipgB1, espG, virA, ipgB2, ipgD, sopB	S. enterica, E. coli, B. pseudomallei, B. mallei, B. thailandensis
22	Vibrio cholerae infection	Type II secretion system	ctxA, ctxB, ace, rtxA	V. cholerae*, E. coli*, V. vulnificus, Y. enterocolitica, A. hydrophila
23	Vibrio cholerae infection	Type IV pilus	tcpA, tcpB, tcpC, tcpD, tcpE, tcpF	V. cholerae*, C. rodentium, E. cloacae, R. aquatilis, R. sp.
24	Vibrio cholerae pathogenic cycle	Quorum Sensing	luxQ, luxU, qrr, tcpB, K10917, aphA, tcpH, tcpP	V. cholerae*, V. vulnificus, V. fischeri, V. parahaemolyticus, V. sp.
25	Epithelial cell signaling in Helicobacter pylori infection	Type IV secretion system	cag1, cag2, cag1, cag2, cag3, virB11, lvhB11, cag7-cag25	H. pylori*, R. prowazekii, L. pneumophila, R. rickettsii, S. meliloti
26	Epithelial cell signaling in Helicobacter pylori infection	Adhesins	hopC, alpA, hopB, alpB, hopZ, K15846, hpaA, sdbA, sabA	H. pylori*, L. pneumophila*, H. cetorum, H. acinonychis, H. bizzozeronii
27	Pathogenic Escherichia coli infection	Intimate adhesion/Type III secretion system	espG, virA, tir, espF, map, eae, nleA, nleH, espH, tccP, espG2	E. coli*, S. flexneri, S. sonnei, C. rodentium, S. boydii
28	Shigellosis	Motility	icsA, virG, espG, virA, icsB, bopA	E. coli, B. pseudomallei, B. mallei, S. flexneri, S. sonnei
29	Shigellosis	Type III Secretion/Downsteam	ipgB1-2, ipaH9.8, ospE-G, espO, mkaD, sipA-D, ipaA-D, bipB-D, ipgD, sopB	S. enterica*, E. coli*, B. pseudomallei*, B. mallei*, E. tarda
30	Salmonella infection	Type III secretion system/SPI-1 encoded	sipB, ipaB, bipB, sopE, ipgD, sopB, sipA, ipaA, sptP, yopJ	S. enterica*, Y. pestis, B. pseudomallei, E. coli*, B. mallei*
31	Pertussis	Type IV secretion system	ptxD, ptxB, ptxE, ptxC, ptxA	S. enterica*, B. pertussis, Y. enterocolitica, B. bronchiseptica, B. parapertussis
32	Legionellosis	Adhesion/Cell entry	sdeA, laiA, rtxA1, rtxA, enhC, lpnE	L. pneumophila*, C. burnetii, L. longbeachae,
33	Legionellosis	Dot/Icm secretion system	ralF, lidA, sidC, legK1, lgt1_2_3, sidI,M, FlepB, vipA,D,E, sdbA, sdcA, drrA, lubX	L. pneumophila*, L. longbeachae,
34	Staphylococcus aureus infection	Colonization/MSCRMMs	clfB, isdA, sdrC_D_E, sasG	S. aureus*, C. pseudotuberculosis, S. epidermidis, S. pseudintermedius, E. casseliflavus
35	Staphylococcus aureus infection	Surface proteins	spa, sbi, clfA, sak, scn, scin, fib, efb, chp, chips, flr, flipr	S. aureus*, S. lugdunensis*, S. pseudintermedius, B. thuringiensis, S. carnosus

The symbol * identifies those genera for which the circuit shown was also linked to pathogenicity via within-genus comparison

**Table 4:** Nonpathogen-linked gene circuits in bacterial strains. The columns identify pathways, circuits, nonpathogen-linked orthologs within the circuit; and genera expressing the circuit

	Pathway	Circuit	Orthologs	Genera
1	Steroid biosynthesis	Squalene to 24,25-Dihydro-lanosterol	SQLE, ERG1, E5.4.99.7, LSS, ERG7, DHCR24	C. coralloides*, F. taffensis, M. capsulatus*, M. alcaliphilum, S. aurantiaca
7	Arginine and proline metabolism	N-Acetyl-glutamate - N-Acetyl-ornithine	argB, ARG56, argC, lysY, ARG56, E2.6.1.11, argD, argD, lysJ	E. coli*, S. enterica*, S. aureus*, L. monocytogenes, M. tuberculosis*
3	Biosynthesis of 12-, 14- and 16-membered macrolides	Propanoyl-CoA to Erythromycin A	E2.3.1.94, eryF, eryBV, eryCIII, eryCII, eryG, eryK	F. alni, S. erythraea,
2	Lysine biosynthesis	L-2-Amino adipate to Pyrrolysine	lysX, lysZ, lysY, lysJ, E2.4.1.173, pylB, pylC, pylD	T. thermophilus*, P. mucilaginosus, C. sp., D. hafniense, M. ruber
5	Glycine, serine and threonine metabolism	Choline - Glycine	codA, gbsB, BHMT, DMGDH, SARDH	B. amyloliquefaciens, B. subtilis, S. meliloti, R. sphaeroides, B. licheniformis
6	Arginine and proline metabolism	Creatine Pathway	GAMT, E2.7.3.2, E3.5.3.3, E3.5.1.59, hyuA, hyuB, E3.5.4.21	H. pylori*, P. putida, C. sp., A. mediterranei, G. sp.
11	Type I polyketide structures	Rifamycin B	rifA, rif14, rifB, rif20, rifC_D, rifE, rifF, asm9, rif19	A. mediterranei*, F. sp., A. mirum, M. aurantiaca, M. sp.
4	Biosynthesis of 12-, 14- and 16-membered macrolides	2-Methylbutanoyl to Avermectin	aveA, aveE, aveF, aveD, aveBI	S. avermitilis.
8	Streptomycin biosynthesis	scyllo-inosamine	stsE, strB1, E2.4.2.27, strK, K12570, aphD, strA	S. griseus
9	Type I polyketide structures	Erythromycin A	eryK, E2.3.1.94, eryF, E2.3.1.94, eryCIII, eryCII, E2.3.1.94, eryBV	F. alni, S. erythraea.
10	Type I polyketide structures	Avermectin A1a	aveA, aveBI, aveE, aveF, aveD	S. avermitilis,
12	Type I polyketide structures	Myalamid S	mxaB, mxaE_D, mxaF	G. violaceus, H. ochraceum, H. aurantiacus, M. xanthus, N. punctiforme
13	Biosynthesis of type II polyketide backbone	7,9,12-Octaketides	actI1, actI2, actI3, actIII, actVII	A. mediterranei*, F. sp., A. missouriensis, C. acidiphila*, C. epipsammum
14	Biosynthesis of type II polyketide products	7,9,12-Octaketides - Actinorhodin	actVI1, RED1, actVI3, actVI2, actIV, actVI4, actVA6, actVIA, actVA5, actVB	A. mediterranei*, F. sp., M. abscessus, S. cattleya, A. ferrooxidans
15	Serotonergic synapse	Signal transduction	PLA2G4, CPLA2, ALOX5, PTGS2, COX2, PTGS2, COX2	R. sphaeroides*, C. coralloides, G. obscurus, M. nodulans, M. sp.
16	Insulin signaling pathway	Glycogenesis/ antilipolysos	GYS, CALM, PHKA_B, PRKAR	S. sp., C. sp., A. sp., B. coagulans, C. sp.
17	Chemical carcinogenesis	Azo dyes/Liver cancer/Bladder cancer	PTGS2, COX2, CYP1A1	R. sphaeroides*, G. obscurus, M. mediterranea, M. nodulans, M. sp.

The symbol * indicates those genera for which the circuitry was linked to synergy via within genus comparison

#### Gene clusters more common in pathogenic strains

**Fig. 6:**
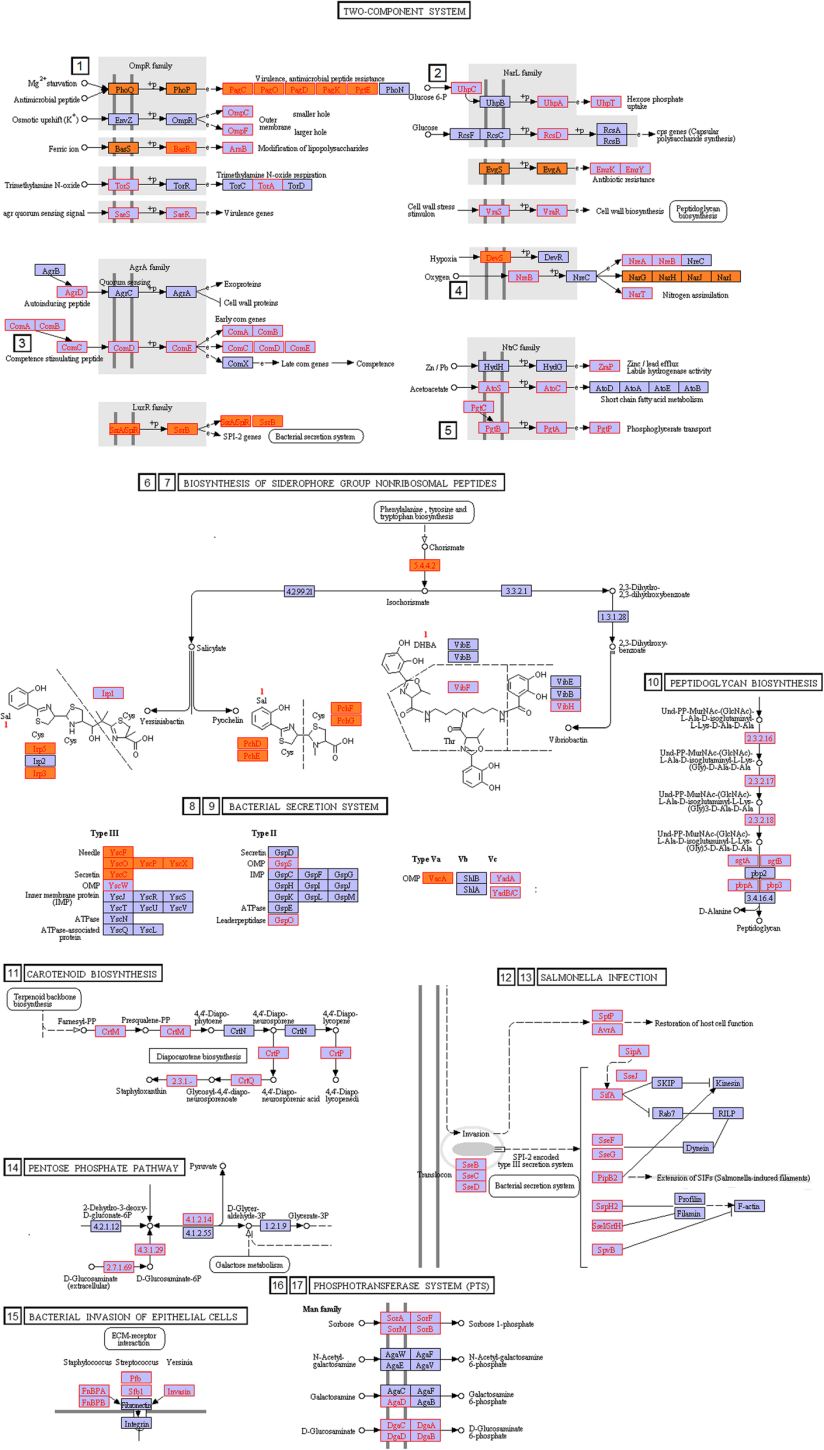
Examples of gene circuitry containing pathogen-linked ortholog clusters in KEGG reference pathways. Orthologs with *PA* > 4 but not present in VFDB were shaded in pink whereas orthologs with *PA* > 4 and also in VFDB in orange. The numbers indicating specific circuitry correspond to their identification numbers in Table 3

The Table 3 presents a set of gene circuits with the ortholog genes linked to pathogenicity and also indicates the pathway to which the gene circuits belong. Along with Table 3, comes Fig. 6, in which the wiring diagrams for the gene circuitry are shown in the form of cutouts from the KEGG Reference pathways. The circuits in the figure have the same ordering number used in Table 3. Note also that the actual circuits contain orthologs not only pathogen-linked (shown in pink and orange) but also others, some preferentially found in pathogenic strains and others more ubiquitous. The p value through hypergeometric test for a bacterial strain containing at least one pathogen-ortholog in a circuit shown in Table 3 was less than 0.01.

**Fig. 7:**
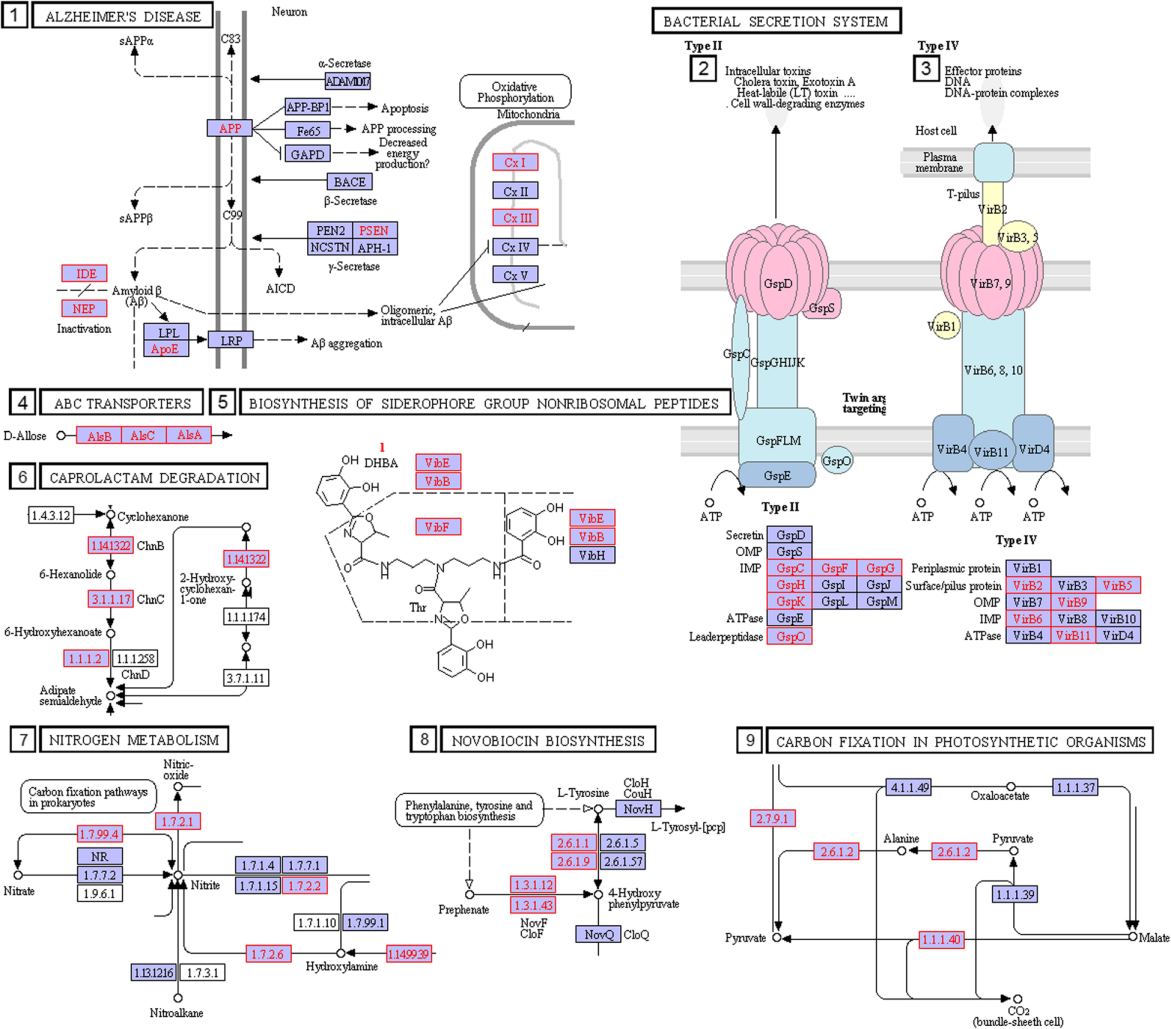
Examples of gene circuitry in KEGG Reference pathways, which are linked to pathogenicity via within-genus comparison. The orthologs linked to pathogenicity in these circuits are shaded in pink. The numbers indicating specific circuitry correspond to their identification numbers in Additional file 5
  - Another pathogen-linked gene circuitry that comes out in genera-specific comparisons is the Amyloid B and Mitochondrial Dysfunction circuitry in the KEGG Alzheimer’s pathway (Additional file 5). The pathogen-linked orthologs in this circuitry (*UQCRFS1*, RIP1, petA, MME, IDE, ide, CALM, *NDUFV2*) are found in 16 of the 17 genera under consideration. This observation suggests the diversity of a bacterial infection that could be linked as a possible modulator of the Alzheimer’s disease [62–64].

The circuitry in Table 3 and Fig. 6 falls into the following categories:

- Gene circuits for bacterial secretion and invasion pathways:
  - The *type III* bacterial secretion system pathway mediates toxin and protein delivery to host cells. Table 3 shows the existence of multiple clusters of pathogen-linked orthologs in this pathway. Consistent with our findings, the type III pathway is listed in the literature as modulating pathogenic interactions with host organisms including animals and plants [29–32].
  - Also shown in Fig. 6 are examples of pathogen-linked circuits in the secretion system. One such circuit contains pathogen-linked orthologs *yscF*, *yscO*, *yscP*, *yscX*, *yscC*, and *yscW*. Subsets of pathogen-linked orthologs of the cluster are present in 242 pathogenic and 106 nonpathogenic strains, resulting in vanishing p values in hypergeometric test. Moreover, the bias towards pathogenicity increases dramatically with increasing number of pathogen-linked orthologs in this cluster in the genome of the bacterial strain.
  - Another secretion-linked pathway is that of *type IV* gene circuit, for which some of its genes exist in both pathogenic and nonpathogenic strains. The circuit functions in translocation of DNA and protein substrates to target cells via direct cell-to-cell contact. In our study, the complete circuit is preferentially present in pathogenic strains. Consistent with these observations, recent investigations uncovered a role for pathogenicity for this circuit [33–35].
- Pathogen-linked gene clusters in the two-component System:
  - The two-component regulatory system is a stimulus–response coupling pathway, which enables bacteria to sense and respond to changes in its environment [36–40]. Membrane-bound histidine kinases are major building blocks of the pathway.
  - These signal transduction systems modulate crosstalk between species within the microbiome. The Table 3contains multiple gene clusters (circuits) in the two-component system containing orthologs linked to pathogenicity: cluster (*devS*, *nreA*, *nreB*, *narT*) involved in hypoxia, oxygen, and nitrogen assimilation; cluster (*uhpC*, *uhpA*, *uhpT*) modulating hexose phosphate uptake; and the cluster (*pagC*, *pagO*, *pagD*, *pagK*, *pgtE*) involved in Mg2+ starvation, and others. See also Fig. 6 for the wiring diagrams of these clusters. Elements of the metabolite assimilation cluster have been linked in the literature to pathogenicity of mycobacterium tuberculosis [41, 42]. The second cluster in the list in the two-component system, mediating hexose phosphate uptake, plays an important role in the sodium-dependent D-glucose transport protein of Helicobacter pylori [43]. This gene circuit is involved in Mg2+ starvation and was shown to play a role in the pathogenicity of Salmonella enterica [44]. Mg2+ starvation is also involved in quorum sensing of Pseudomonas fluorescens [45] and in biosynthesis of complex lipids needed for virulence of mycobacterium tuberculosis [46].
- Metabolic circuits linked to pathogenicity:
  - A metabolic gene circuit whose genes are commonly found in pathogenic strains is the *CMP-Pse metabolism circuit* cluster belonging to the amino nucleotide sugar mechanism. Pathogen-linked ortholog genes in this circuit consist of *pseC*, *pseH*, *pseF*, and *UAP1* (Table 3). This circuit is linked to the synthesis of glycoconjugates, which are typically expressed on the surfaces of pathogenic bacteria. The protein products of the circuit have already been identified as virulence factors in the VFDB database and in the literature [47–49].
  - Nodal elements of the *Peptidoglycan biosynthesis circuit* cluster shown in Table 3 are also preferentially present in pathogenic strains. Pathogen-linked orthologs in this gene circuit consist of the genes *sgtA*, *sgtB*, *femA*, *pbpA*, *femB*, *pbp3*, *femX*, and *fmhB*. Peptidoglycans are polymers consisting of sugars and amino acids forming a mesh scaffold external to the plasma membrane. Recent studies in the literature point to the role of peptidoglycans in the pathogen phenotype of different bacteria [50–52].
  - Sorbose to Sorbose 1-phosphate circuit of the Phosphotransferase (PTS) system also shown in Table 3 contains pathogen-linked genes *PTS-Sor-EIIC*, *sorA*, *PTS-Sor-EIID*, *sorM*, *PTS-Sor-EIIA*, *sorF*, *PTS-Sor-EIIB*, *sorB*. PTS circuit codes a group translocation process present in many bacteria, transporting sugars from the environment into the bacterial cell. The circuit has been linked in the literature to Streptococcus invasion [53].
  - Our statistical computations based on hypergeometric test indicate that the likelihood of pathogenic identification of a strain increases dramatically with increasing numbers of the circuit genes expressed in the strain’s genome. Reflecting this finding, the Table 3 contains 15 gene circuits for which bacterial strains containing at least 75 percent of the circuit elements are always pathogenic. Hence a signature for pathogenicity may be derived from the study of clusters of pathogen-linked orthologs in bacterial strains.
  - Figure 6 presents other examples of ortholog groupings listed in Table 3 and acting in tandem in pathogenic processes. One such circuitry shown in the figure is involved in the biosynthesis of siderophore group of nonribosomal peptides. These are high affinity iron binding compounds [54] and were found to play an important role in virulent bacterial infections [55]. Also shown in the Figure is pathogen associated ortholog circuit clusters crowding the bacterial secretion system not discussed above in detail. As noted in the literature, the secretion system facilitates transport, injection, and release of effector compounds including enzymes, and toxins in bacteria [56, 57].
- Additional pathogen-linked circuitry identified through comparisons of genomes belonging to the same genera:
  - Additional circuits linked to pathogenicity could be identified using within-genera genome comparisons. We have conducted comparisons of ortholog contents of strains belonging to the same genera for the seventeen genera with most number of strains in our dataset, shown in Table 1. Again, the clusters of pathogen-linked orthogs forming on KEGG reference pathways were identified. However, in this case, we relaxed the pathogen-linkage evaluation from PA > 4 to PA > 2 since genomes belonging to the same genera are more or less similar. In addition, we are looking here for circuitry common across genera.
  - Results of these computations are presented in Additional file 5, identified in rows 1 to 21 for pathogen-linked circuits. The table shows not only the circuitry but also the genera associated with the specified circuitry. The circuit clusters most common across genera in this Table lie in the pathways for glycine, serine and threonine metabolism and sulfur metabolism (Fig. 7). These pathways have been implicated in playing important roles in pathogenicity [58–61]. Also in this category, is the gene circuit in Additional file 5 row 1 linked to Alzheimer's disease via Amyloid B and Mitochondial Disfunction.

#### Gene circuits found in nonpathogenic strains

**Fig. 8:**
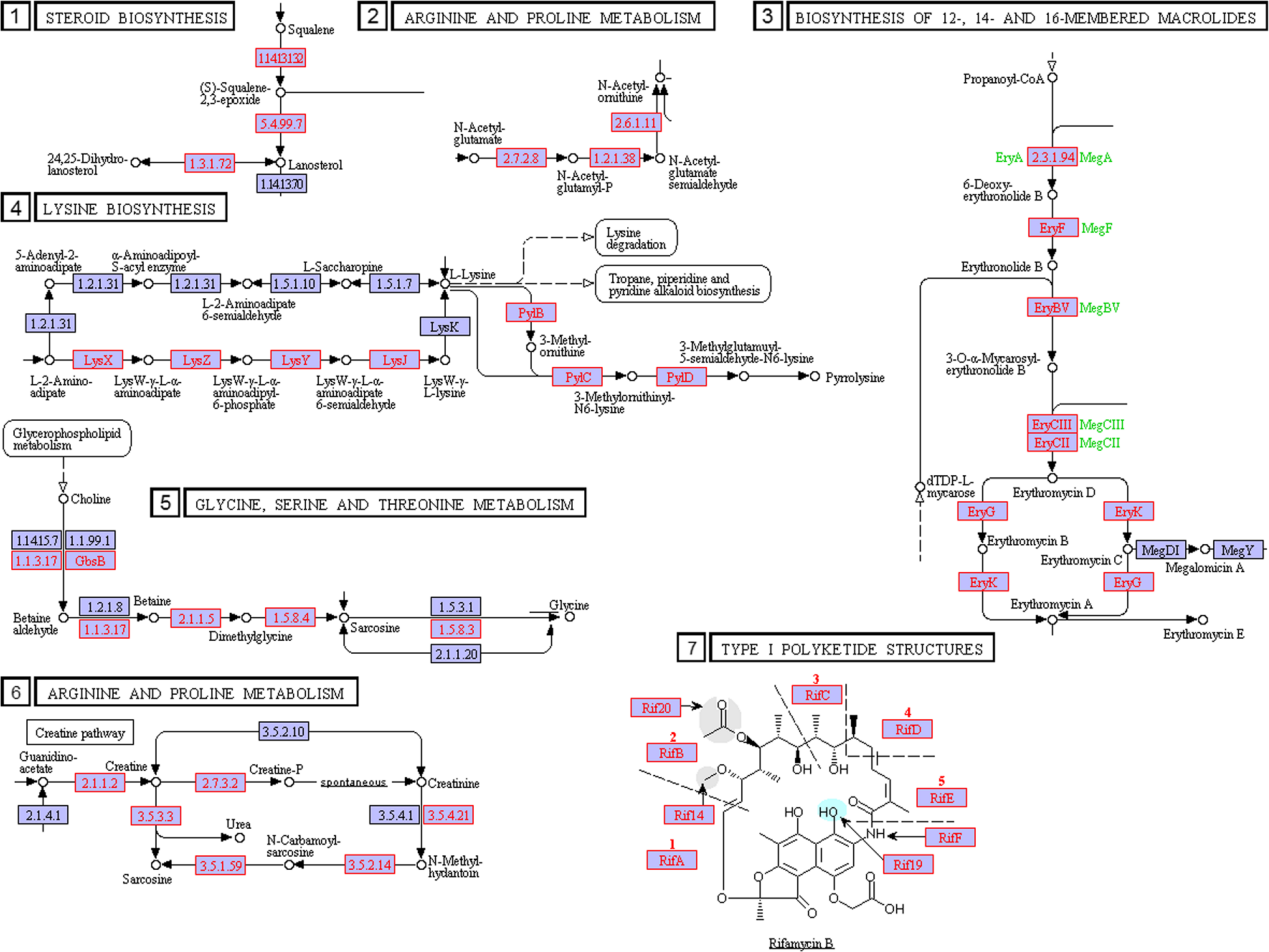
Examples of gene circuitry containing nonpathogen-linked ortholog clusters in KEGG reference pathways. Orthologs with *PA* < 1/4 are shaded in pink. The numbers indicating specific circuitry correspond to their identification numbers in Table 4

Circuits linked to nonpathogenicity are shown in Tables 4 and Additional file 5, respectively, for across genera and within genera comparisons. Our detailed results shown in Table 4 and Fig. 8 are summarized below.

Antibiotics and metabolite producing circuits:

- Presence of clusters of nonpathogen-linked orthologs in metabolic circuits such as steroid biosynthesis, arginine and proline metabolism, and the Insulin signaling pathway indicate the importance of these pathways in establishing synergy with the host in all the major genera considered (Table 4). Some of the orthologs in the nonpathogen-linked bacterial gene circuits have orthologs in the human. Other synergy circuits in Table 4 are involved in radiation survival [65]. KEGG reference metabolic pathways contain large numbers of nodes creating thousands of clusters for bacterial species, and hence the relative lack of literature for some of the clusters shown in Table 4.
- The polyketide circuit shown in Fig. 8 and presented in Table 4 facilitates the synthesis of common antibiotics [68]. Polyketides are complex organic compounds, which are highly active biologically. Many pharmaceuticals are derived from or inspired by polyketides. In addition to the polyketide circuits, the circuit shown in Fig. 8 composed of scyllo-inosamine orthologs (*stsE*, *strB1*, *E2.4.2.27*, *strK*, *K12570*, *aphD*, *strA*) is involved in the biosynthesis of streptomycin and similar anti-mycobacteria antibiotics. Recent studies show bacterial virulence factors in type 3-secretion pathway as targeted by virulence inhibitors such as those illustrated in Fig. 8 [69]. Also shown in the figure is the *one-carbon pool by folate pathway*, activating one-carbon units for biosynthesis [70]. It plays a major role in amino acid metabolism [71]. It has been shown to affect proof reading of DNA replication, DNA methylation, and chromatin structure [72–74]. The list for commensal circuitry presented in Table 4 is not complete, but representative of the diversity of commensal circuits found in bacterial strains.
- Genera-specific genome comparisons reveal additional circuitry clusters found almost exclusively in nonpathogenic bacterial strains. Shown in Additional file 5 in rows 22 to 24 are clusters for benzoate degradation, and the cluster for dopamine circuitry in Isoquinoline alkaloid biosynthesis. Benzoate degradation is an important factor in reducing drug-induced toxicity [66]. It is not clear how dopamine inducing bacterial gene circuits drive synergy with the host, yet modulations in dopamine circuitry in bacteria was previously linked to Alzheimer’s disease stage progressions via Borrelia infection [67].

## Discussion

Pathogenicity is context dependent. A survey of the literature indicates many dimensions of complexity in defining and investigating pathogenic behavior of bacterial strains and microbiomes in relation to host [75–78]. Consider a microbiome composed of thousands of bacterial strains. The combined genome is large, containing millions of genes. It is clear in this scenario the need for obtaining a signature for pathogenic or commensal phenotypes for dimension reduction. This study begins with such a task, namely deducing a list of genes which are highly abundant in pathogenic and relatively absent in commensal strains and vice versa. Two distinct datasets were used in our analysis: (a) list of bacterial strains observed to be pathogenic at least in a context dependent manner and (b) bacterial genomes annotated with ortholog genes. The literature search yielded 949 decoded bacterial genomes deemed as pathogenic and another 1578 as nonpathogenic or commensal due to lack of evidence in the literature for pathogenicity. Additional file 1, presenting the list of pathogenic strains along with literature-curated evidence for each, will prove to be a useful resource for microbiology.

The ortholog contents of genomes in KEGG Orthology (KO) Database were recently used in developing an algorithm (PICRUSt) for analysis of genes in microbiomes in health and disease [79]. In the present case, KO database yielded two large matrices with columns identifying pathogenic or nonpathogenic strains and rows representing 7194 orthologs present in at least one bacterial strain. We created similar matrices for within genera comparison of pathogenic and nonpathogenic strains. From this point on, we could compare the two matrices and identify, for each ortholog, the relative abundance in pathogenic and nonpathogenic strains. The resulting ortholog based files compiled in Additional file 2 is easy to use, as for each ortholog, we provide its NCBI identified symbol, full ortholog name, and KEGG ID. In essence, the focus on orthology enabled genome comparison at a meta-scale, and enabled us to integrate discrete pieces of data in the literature into a system wide portrait.

Statistical enrichment processes we employed allowed us to investigate antibiotic resistance within the context of pathogenicity. Specifically, we considered pathway enrichment of orthologs present in 268 antibiotic resistant bacterial strains with respect to the 681 pathogenic strains with no documented antibiotic resistance. Some of the orthologs associated in the literature with antibiotic resistance [80] were indeed preferentially abundant in antibiotic resistant strains. Others were not but they co-localized with orthologs highly enriched in antibiotic resistant strains in cellular pathways. The pathways enriched by orthologs abundant in antibiotic resistant strains included P13K-Akt signaling pathway of eukaryotic hosts. The effector bacterial orthologs in this pathway divert host cell signaling pathways to the benefit of the pathogen and target kinase signaling cascades present in P13K-Akt [81], resulting in pathogenic infections [82]. The other most enriched pathway in antibiotics resistant bacerial strains was peptidoglycan biosynthesis, previously linked to biofilm production [83]. The pathway mediating the metabolism of Sphingolipids, a class of lipids, was occupied by orthologs found in antibiotic resistant bacteria. Sphingolipids play significant roles in membrane biology and provide many bioactive metabolites that regulate cell function [84]. It has already been linked in the literature to antibiotic resistance [85, 86].

Pathway enrichment protocols provided us with an overall portrait of ortholog sets linked either to commensal or pathogenic behavior to the host. Additional annotation was needed however in order to get a signature for synergy or pathogenicity. Visually, when we projected pathogen- or synergy-linked ortholog lists onto KEGG pathways, we could see orthologs from our lists forming multiply connected clusters along the bacterial pathways. Thus, our next task was identifying circuits along the pathways that contained such clusters and then to conduct extensive literature searches to annotate our discoveries.

The circuits identified included toxin-producing units for pathogenic strains and the gene circuits synthesizing antibiotics for commensal strains. We observed that the list of circuitry linked to synergy contained among others, biosynthesis modules of type II polyketide products, such as Erythromycin, and Doxycycline. Other examples of nonpathogen-linked ortholog clusters in gene circuits could be found in VEGF signaling pathway and in metabolic pathways.

The ortholog modules highly abundant in pathogenic bacteria included gene circuits found in bacterial secretion mechanism type III. Also in this category were gene circuits involved in peptidoglycan biosynthesis. In general, pathogen abundant orthologs were statistically enriched in KEGG pathways for pathogen interactions and in signaling pathways. Comparison of pathogenic strains with nonpathogenic strains of the same genera identified *one carbon pool by folate pathway* as highly abundant in pathogenic strains. This pathway mediates the activation of one-carbon units in the biosynthesis and metabolism of amino acids. It affects DNA methylation as well as DNA replication. Recent research implicates it in folic acid-mediated degeneration of the brain–blood barrier of the host [87]. In addition, our system approach identified a bacterial gene circuit whose genes are preferentially found in pathogenic strains and linked in the literature to Alzheimer’s disease. We find the gene clusters in this circuit to be abundant in a diverse set of genera, possibly providing new avenues of research for linkage between bacterial infections and Alzheimer’s disease.

The work presented here is a first draft of an encyclopedia for bacteria – host interactions. The study uncovers identity of ortholog clusters as possible signatures of pathogenicity or synergy in a mixture of bacteria. We show that gene clusters formed on bacterial pathways to be much stronger determinants of pathogenicity than a list of virulence and/or anti-virulence factors. The signature we derive in the form of gene circuits may not be complete as our results relied on the decoded genomes of bacterial strains currently available in the literature. Moreover, our reliance on the KEGG reference pathways in identifying clusters of orthologs preferentially found in pathogenic and nonpathogenic bacteria introduces additional limitations due to incompleteness of the KEGG pathway models. Nevertheless, the gene circuitry signature we discovered for synergy and pathogenicity is comprehensive enough to derive from it biomarker clusters identifying pathogenic phenotypes in bacterial strains isolated or in a mixture.

## Conclusions

This study presents a system approach for identifying gene clusters either preferentially present or absent in pathogenic bacterial strains. The study utilized 2527 fully sequenced bacterial strain genomes available in the public domain. Literature search identified 949 of these genomes to belong to strains with demonstrated pathogenic potential. Ortholog abundance comparisons between pathogenic and nonpathogenic strains within and across genera revealed signatures for pathogenic and commensal bacteria. Known virulence factors were highly enriched in the genomes of pathogenic strains.

Projection of ortholog gene signatures onto cellular pathways revealed gene circuits linked either to synergy or pathogenicity. The *pathogenicity related gene circuits* included those in bacterial *two-component* system, biosynthesis of *siderophores*, and *one-carbon pool by folate pathways*. Circuits belonging to *shingolipid* metabolism, *P13-Akt pathway*, and *tRNA* synthesis were particularly enriched with orthologs preferentially expressed in *antibiotic-resistant bacteria*. Genes preferentially expressed by *nonpathogenic bacteria* also formed circuits, among which were those linked to the synthesis of antibiotics. The study comprises an important step towards addressing crosstalk between host, virus, prokaryotes, and environment using a system approach [88–90].

## Methods

### Annotation of bacterial strains exhibiting pathogenicity

The list of pathogenic bacterial strains was obtained via literature search using three complementary and partially redundant approaches. First, we text mined the Kyoto Encyclopedia of Genes and Genomes (KEGG) database [91, 92] for pathogenicity label for all decoded bacterial strains in KEGG. Second, we identified as pathogenic those bacterial strains with pathogenic citation in at least one of the following web tools: Virulence Factor Database (VFDB) [2, 6, 9], High-quality Automated and Manual Annotation of Proteins (HAMAP) [93], and the Interactive Atlas for Exploring Bacterial Genomes (BacMap) [94]. Third, we text mined PUBMED article abstracts with a) the name of the bacterial strain and b) one of the pathogen-related code words (pathogen, virulence, pathogenic, virulent). All hits were then verified for pathogenicity by reading the articles. We labeled bacterial strains as nonpathogenic if the process described above did not yield evidence for pathogenesis. We have used the Antibiotic Resistance Database (ABRD) [25] to identify *antibiotic resistant bacterial strains* within the set of pathogenic bacterial strains.

### Orthology content of decoded bacterial strains

KEGG programming interface was used to obtain gene orthology information for the bacterial organisms found in KEGG. The individual organism orthology content was translated into a logical content vector that describes the orthology information for that particular organism in binary form, “1” meaning that the ortholog is present and “0” meaning it is absent, with respect to all collective orthologs found in the KEGG repository. The individual feature vectors were than accumulated into a logic matrix, with rows representing the presence/absence of orthologs for each decoded bacterial strain. The resulting array contained 2527 sequenced bacterial strain genomes potentially expressing 7194 orthologs. The number of genes that were accounted for in this array totaled to over 3.2 million. The logical array was then split into pathogenic vs. non-pathogenic logical arrays with equal number of columns representing the orthologs.

### Abundance scores for orthologs in bacterial strain genomes

An abundance score for each ortholog was generated for both the pathogenic and nonpathogenic bacterial strain arrays based on the sum of all elements in the ortholog column vector divided by vector length (number of bacterial strains). These scores were denoted as *Ap* and *Anp* for pathogenic and nonpathogenic strains, respectively. They represent the fraction of genomes expressing the ortholog in pathogenic and nonpathogenic logical matrices. Next we defined a pathogenicity abundance score as *PA* = *Ap / (Anp + 0.0001).* If *Anp* turned out to be “0” for that ortholog, meaning that ortholog was absent in nonpathogenic bacteria, the equation yields PA as equal to Ap / 10^−4^. The list of orthologs could then be ordered with respect to the *PA* value, creating a histogram.

The comparisons of contents of genome orthology of bacterial strains belonging to the same genera are identical to the one for all the 2527 content comparison described above. For this operation, we used only strains belonging to the genera shown in Table 2, one genus at a time. Also, in this case we reduced the cutoffs for pathogen association and defined orthologs as pathogen-abundant for *PA* > 2, and nonpathogen abundant for *PA* < 1/2. This was necessitated by the relative similarity of genomes within a genus. However, to compensate, we studied only those clusters in KEGG Reference pathways abundant in pathogen or nonpathogen strains in multiple genera.

### Statistical enrichment of cellular pathways with pathogenic and nonpathogen-abundant orthologs

We identified orthologs as pathogen exclusive if they were expressed in some of the pathogenic strains but not at all in nonpathogenic strains. Nonpathogen exclusive orthologs were similarly defined. We defined orthologs as pathogen-abundant for *PA* > 4, and nonpathogen abundant for *PA* < 1/4. These were also called pathogenic orthologs and vice versa.

For KEGG cellular pathway enrichments, we created a score matrix, similar to that described in the making of the orthology database. Next, the enrichment was carried out via hypergeometric test [63, 95] using total number of orthologs in bacterial strains as the population size, pathogenic orthologs as the number of success states in the population; number of orthologs in the pathways as the number of draws; and the number of pathogenic orthologs in the pathways as the number of successes in the draw.

Subpathways containing pathogenic ortholog circuits were identified via manual KEGG orthology mapping and screening for clustering. Cellular pathways were drawn using the KEGG web tool for pathways. Although the cutoff values for *PA* for generating pathogenic and nonpathogenic ortholog lists appear arbitrary, they capture the tails of the *PA* distribution for orthologs in the thousands of decoded genomes under consideration. Preliminary studies involving the perturbation of the cutoff did not yield variation in the enriched pathways.

### Ortholog clusters relevant to pathogenicity in KEGG reference cellular pathways

For this purpose, we mapped the list of pathogen-abundant and nonpathogen abundant orthologs to all available KEGG Reference pathways, and manually curated the clusters (sub circuitry) they form. The criterion for cluster was the minimum of three orthologs from the list to be multiply-connected with each other and have connections to other neighboring orthologs. Results shown in Table 3 to Additional file 5 indicate only the gene symbols of those abundant in pathogenic or vice versa. As presented in these tables, most clusters contained many more orthologs then three. However, the Reference pathways typically contain genes not found in all strains and hence in almost all cases we studied 75 % presence of pathogene-abundant orthologs in a strain as indicating that the strain was deemed pathogenic in the literature. The p values listed in these tables were obtained using hypergeometric test based on the following scheme: a) there are 949 strains deemed pathogenic in a pool of 2527 strains, and b) among M strains, m cluster orthologs were found only on N strains. In this set up, M, N, and m are positive integers.

## Additional files

Additional file 1:
**List of 949 decoded bacterial genomes present in the KEGG web platform deemed as pathogenic along with literature citations for pathogenicity and antibiotic resistance phenotype.** (XLSX 51 kb) Additional file 2:
**List of 1578 decoded bacterial genomes present in the KEGG web platform with no present citation implicating them as pathogenic.** (XLSX 54 kb) Additional file 3:
**Bacterial strain orthology logic array indicating the presence of given orthologs (7194 of them) in the 2527 bacterial strain genomes considered in this study.** (RAR 1397 kb) Additional file 4:
**List of 7194 orthologs ordered according to pathogen association score** **PA.** The orthologs shaded in pink represent ones present in VFDB. (XLSX 570 kb) Additional file 5:
**Pathogen- and nonpathogen gene circuits identified via within genera comparisons.** The genera exhibiting a pathogen-linked circuit are painted in pink whereas nonpathogen-linked circuits are painted in green.

## Abbreviations

ARDB: Antibiotics resistance genes database
KEGG: Kyoto encyclopedia of genes and genomes
PA: Pathogenicity index
VEGF: Vascular endothelial growth factor
VFDB: Virulence factor database
