# Supplementary material for: Encyclopedia of bacterial gene circuits whose presence or absence correlate with pathogenicity – a large-scale system analysis of decoded bacterial genomes
Source: BMC Genomics. 2015 Oct 13;16:773. doi: 10.1186/s12864-015-1957-7 (PMC4603813; doi:10.1186/s12864-015-1957-7)
Supplement: Additional file 5: — Pathogen- and nonpathogen gene circuits identified via within genera comparisons. The genera exhibiting a pathogen-linked circuit are painted in pink whereas nonpathogen-linked circuits are painted in green. [file 12864_2015_1957_MOESM5_ESM.docx]

Additional file 5. Pathogen- and nonpathogen gene circuits identified via within genera comparisons. The genera exhibiting a pathogen-linked circuit are painted in pink whereas nonpathogen-linked circuits are painted in green.
